# The effects of psychological safety and employee voice behavior on flight attendants’ mindful safety practices adoption

**DOI:** 10.3389/fpubh.2024.1398815

**Published:** 2024-09-11

**Authors:** Shi Hu, Muhammad Aamir Nadeem, Ji Luo, Xiaobo Yi

**Affiliations:** ^1^School of Economics, Sichuan University of Science and Engineering, Zigong, China; ^2^Quality Enhancement Cell, Faisalabad Medical University, Faisalabad, Pakistan

**Keywords:** psychological safety, employee voice behavior, ethical leadership, traditionality, flight attendants, mindful safety practices

## Abstract

**Introduction:**

Flight attendants, as the front-line staff in the cabin, play a crucial role in improving air travel safety. This research explores how psychological safety affects flight attendants’ adoption of mindful safety practices through voice participation. This mechanism also identifies ethical leadership and traditionality as two moderators.

**Methods:**

A self-reported questionnaire was used to collect data from 621 flight attendants in Chinese private commercial airline companies. PLS-SEM (partial least square structured equation modeling) is used to examine the hypotheses proposed in the present study.

**Results:**

After data analysis, the results reveal that the underlying mechanism covering both mediating and moderating effects through which flight attendants’ voluntary and extra-role safety behavior could be improved.

**Discussion:**

The findings extend the existing literature regarding the antecedents of flight attendants’ mindful safety practices adoption and obstacles to employee voice participation. Managerial implications are also provided in the commercial aviation industry and discussed along with future research directions.

## Introduction

Accidents and near misses are usually caused by employee’s unsafe behavior, like human errors in the aviation industry (1, 2). By monitoring cabin equipment, flight attendants are responsible for inflight service and safety confirmation during the takeoff, flying, and landing processes (3). Along with the vital role of pilots’ safety behavior, flight attendants as front-line employees also play important roles in ensuring flight safety (4). Among human errors in the aviation industry, some errors originate from the overlaps and errors of flight attendants during the flight task (5), indicating the rationality of flight attendants’ safety awareness, and high attention to the cabin surroundings during the flight.

Flight safety always rank as the top priority in the commercial aviation industry because it’s highly related to human life. Moreover, the reputation and public trust of commercial aviation companies tend to be largely affected when an accident happens. Security is always placed as the priority and first concern among the flight passengers’ expectations and preferences (6). Except for the integration of mindfulness into the pilots’ safety behavior, it is also necessary for the flight attendants to adopt it. Ji et al. (7) said that flight attendants can perform better safety behavior when they have a high level of safety awareness during the flight task. Therefore, flight attendants need to apply mindful safety practices to their flight tasks by identifying and reacting to the potential hazards promptly during the flight. Furthermore, flight attendants with this initiative and proactive safety behavior attempt to estimate the environment on board during routine flight tasks with awareness and attention (7). Mindful safety practices involve hazard identification, which is the first step in the safety risk management (SRM) process.

**Figure 1:**
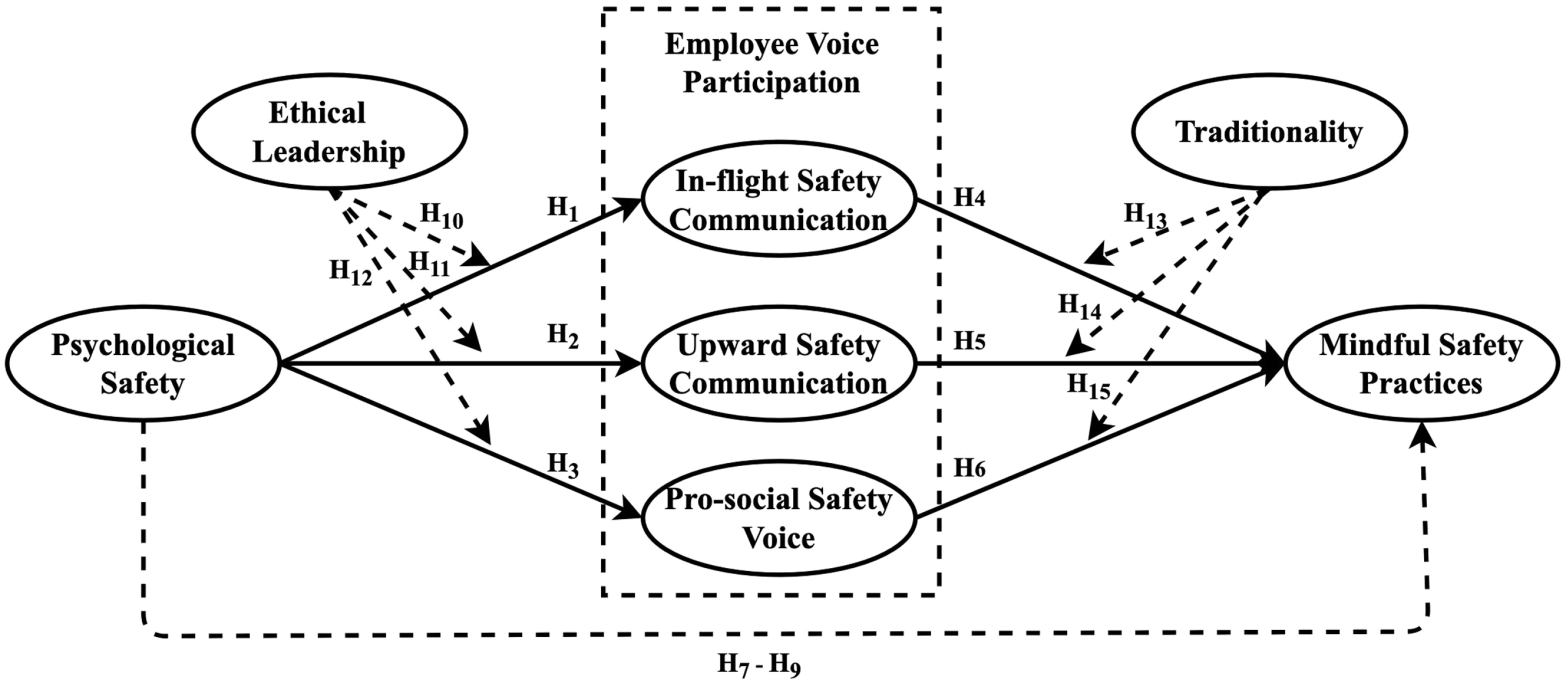
Research framework.

The current study provides additional insights concerning the mindful safety practices conducted by flight attendants in the Chinese commercial aviation industry. Less is known about how flight attendants’ mindful safety practices are prompted through voice behavior in the commercial aviation industry. Safety voice research has been discussed in the health sector, which still lacks enough evidence about the factors that could promote or hinder safety voice behavior in high-risk industries (8). Furthermore, less attention has been paid to examining the integration of voice participation with cabin crews’ proactive safety behavior (mindful safety practices) from the Chinese traditional perspective. This research aims to understand the mechanism underlying the flight attendants’ mindful safety practices from the psychological, leadership, and organizational approaches. The following three major questions are addressed: 1. Does employee voice participation indirectly affect mindful safety practices? 2. Does ethical leadership strengthen the relationships between psychological safety and employee voice participation? 3. Does traditionality weaken the relationships between employee voice participation and mindful safety practices adoption? To answer these three research questions, a research framework is proposed after a careful literature review, as shown in Figure 1.

## Theoretical foundation and hypotheses development

### Theoretical foundation

#### Conservation of resources (COR) theory

Conservation of Resources (COR) theory aims to understand employee motivation and stress in the organizational context. COR theory highlights the importance of various types of resources from organizations, leaders (condition resources), and employees (personal resources). Personal resources refer to psychological safety, while condition resources indicate ethical leadership in this research. There is a need for individuals and organizations to protect against resource losses, recover from resource losses, and gain more resources through resource investments (9). It is stated that supervisory support could lead to a motivational process (10). Moreover, traditionality is identified as a boundary condition to further explore whether individual voice behavior can be weakened to facilitate mindful safety practices. It suggests that different levels of resources should co-exist and maximize their applications to motivate individuals (9). According to COR theory, ethical leadership as a condition resource is associated with the mediating mechanism between psychological safety and the adoption of mindful safety practices.

#### Social exchange theory (SET)

Social exchange theory states that if one party acts in ways that could benefit another party then an implicit obligation is generated, which causes further reciprocity from the beneficial party to the initial party (11). Based on social exchange theory (SET), it hypothesized that employees who perceive that their leaders are honest, trustworthy, and fair, employees’ positive reciprocity would come (12). Employee voice participation is a reciprocal response to the supervisors and organizations after feeling cared for and concerned by the leaders and organizations. Furthermore, under SET, employee voice behavior could be triggered as positive reciprocity when they perceive their leaders are supportive, moral, caring, open, and concerned.

### Hypotheses development

#### Psychological safety and employee voice behavior

Psychological safety indicates the individuals’ beliefs and concepts that their behavior (new ideas, error reports, asking for help, feedback, and questions) would not get negative responses or reactions from their colleagues and managers. Employees with psychological safety would give expressions for organizational improvements naturally and necessarily without the fear feeling of potential loss and risks.

The necessity for encouraging flight attendants to conduct comprehensive safety voice behavior is due to their close interactions with diverse groups (pilots, passengers, and pursuers) and direct observations of the cabin as front-line employees (13). Voogt et al. (14) stated that the main concern in employee voice behavior is to express their ideas or suggestions safely and validly with others’ respect and recognition. This study hypothesizes flight attendants with psychological safety are more likely to conduct voice behavior during flight tasks. Therefore, psychological safety is identified as a critical precondition and psychological status for employees’ speaking up behaviors as a form of organizational citizenship behavior (OCB) (15). Thus, the following hypotheses are proposed:

*H1*: Psychological safety is positively related to in-flight safety communication.

*H2*: Psychological safety is positively related to upward safety communication.

*H3*: Psychological safety is positively related to pro-social safety voice.

#### Employee voice behavior and mindful safety practices

Mindful safety practices are defined as the ability to be aware of critical factors in the environment and to act appropriately when dangers arise (16). Mindfulness has the characteristics of treating failures seriously, being aware of the current situations, continuous adjustments, bouncing back, error prevention, error anticipation, and seeking competent experts without hierarchy status consideration (17). Voice behavior is defined as employees expressing their ideas or suggestions or wishing to improve the organization or unit (18). Additionally, front-line employee voice behavior contributes to organizational improvements through constructively expressing ideas and suggestions about work. This study aims to identify if the employees with voice participation are more likely to execute mindful safety practices. The concepts of mindful safety practices are necessary to embed in the flight attendants’ daily work routine, especially under unexpected and urgent situations during the flight task. Although Ji et al. (7) regarded mindful safety practices as an indicator of flight attendant safety behaviors, this promotive safety behavior still gains limited attention. However, there is a lack of more detailed knowledge about how employee voice behavior is related to organizational safety promotion, for example, mindful safety practices. Thus, integrating mindful safety practices into flight attendants’ safety performance must be explored. In this research, in-flight safety communication, upward safety communication, and pro-social safety voice of flight attendants are all identified to gain a comprehensive picture of flight attendants’ voice behavior. As such, the following hypotheses were developed:

*H4*: In-flight safety communication is positively related to mindful safety practices.

*H5*: Upward safety communication is positively related to mindful safety practices.

*H6*: Pro-social safety voice is positively related to mindful safety practices.

Moreover, the subsets of mindful safety practices also require the watchman role of the employees, which needs to warn colleagues if necessary and also stop any behavior that may harm their colleagues (16). This implies the necessity of considering employee psychological safety to ensure their voice participation and further facilitate mindful safety practices adoption. The reason is that employees with psychological safety have no fear of raising voices and concerns about their colleagues and organization. So, psychological safety is identified as an antecedent of adopting mindful safety practices through voice participation in this research context. Thus, the following hypotheses are proposed:

*H7*: In-flight safety communication mediates the positive relationship between psychological safety and mindful safety practices.

*H8*: Upward safety communication mediates the positive relationship between psychological safety and mindful safety practices.

*H9*: Pro-social safety voice mediates the positive relationship between psychological safety and mindful safety practices.

Leaders and colleagues exert implicit and inevitable impacts on the employees through their work behavioral preferences, such as employee voice participation encouragement (19). Airline department managers should adopt a suitable leadership style to motivate flight attendants to conduct voice behavior. Therefore, ethical leadership as the contextual factor that could promote employee voice behavior needs to be addressed, especially in the high-risk industry. In this research, we shed light on the moderating effect of ethical leadership, underlying the relationships between psychological safety and employee voice participation.

As such, the following hypotheses were developed:

*H10*: Ethical leadership strengthens the relationship between psychological safety and in-flight safety communication.

*H11*: Ethical leadership strengthens the relationship between psychological safety and upward safety communication.

*H12*: Ethical leadership strengthens the relationship between psychological safety and pro-social safety voice.

This research identifies the antecedents that could facilitate or impede the employee’s voluntary voice behavior in the aviation industry. This research attempt may bridge the gap in the literature by discovering the underlying different motives for flight attendants’ voice behavior. Traditionality hinders employees from challenging the prevalence and proposing different ideas. In the Chinese cultural context, organizations usually have a culture of silence to avoid any interpersonal relationship risks and uncertainty. Employees in this cultural context tend to express agreements rather than disagreements or even their real thinking [Van (20)]. In this respect, traditionality makes the employees benefit less from the psychological safety to participate actively in voice behavior, impeding the implementation of mindful safety practices facilitation. It is of interest to examine the employee voice participation in the context of cultural characteristics, like China. Employees usually estimate the pros and cons before adopting the voice behavior (21), especially in a cultural context with high traditionality. Therefore, it is necessary to explore voice participation in the Chinese cultural context. In the current research framework, it is assumed that traditionality weakens the positive influence of employee voice behavior on mindful safety practices conduction. Therefore, the following hypotheses are proposed:

*H13*: Traditionality weakens the indirect effect of psychological safety on mindful safety practices via in-flight safety communication.

*H14*: Traditionality weakens the indirect effect of psychological safety on mindful safety practices via upward safety communication.

*H15*: Traditionality weakens the indirect effect of psychological safety on mindful safety practices via pro-social safety voice.

## Methodology

The quantitative research method is adopted in this research. After the research framework proposition, this section discusses the unit of analysis, sample size, measures, data collection procedure, and data analysis.

### Unit of analysis

The target population of the current study is flight attendants working for Chinese commercial private aviation companies. The respondents in this research contain the job positions of the flight attendant, deputy purser, and chief purser. It is stated that pursers are the direct supervisors of flight attendants, who arrange their flight duties and monitor performance. Additionally, deputy pursers may only supervise certain flight classes, for example, first, business, or economic-level flight classes (13). Judgment sampling is considered non-probability and increases the accurate estimation of the defined population (22). Thus, some criteria are defined as bias control in choosing the suitable participants in this study. The respondents in the current study focus only on the flight attendants (also pursers and deputy pursers) group in Chinese commercial aviation companies.

### Sample size

To gain a suitable minimum sample size, researchers need to consider the model structure, the anticipated significance level, and the expected effect size (23). So, G Power 3.1 software was adopted for minimum sample size calculation. The minimum sample size was 119 calculated by G Power 3.1 software.

### Measures

A self-administered questionnaire was applied for data collection. Two portions comprise the questionnaire. Section 1 collects the demographic profile of the respondents. Section 2 contains the measurement of items for each construct in this research framework. Ethical leadership was measured using a 10-item scale adopted from Brown et al. (24). A sample item is ‘My leader listens to what employees have to say.’ Employee voice behavior consists of 13 items. Among them, 5 items of upward safety communication and 5 items of pro-social safety voice were adopted and adapted from Chen (13). Another 3 items of in-flight safety communication were adopted from Ford et al. (25). In total, there are 13 items measuring employee voice behavior in this research. It is said that both downward and upward communication about safety issues exert vital effects on the prevention, detection, and correction of unsafe work environments (8). Flight attendants’ voice behavior includes in-flight safety communication with aircrew members during the flight, upward safety communication with supervisors, and pro-social safety voice for organizational improvements. The example item of upward safety communication is ‘I feel comfortable discussing safety with the supervisor.’ In-flight communication involves the flow of information between the flight attendants and other cabin crew members during the flight (25). A sample item of in-flight safety communication is ‘In-flight services management responds to the safety concerns of the flight attendants.’ Further, a sample item of pro-social safety voice is ‘I express solutions to safety problems with the cooperative motive of benefiting the cabin safety.’ Moreover, psychological safety comprises 5 items adapted from Edmondson et al. (26), Dar et al. (27), and Edmondson and Lei (28). A sample item is ‘It is safe to take a risk in this organization by expressing different and constructive ideas about safety issues.’ Traditionality was measured by 3 items adapted from Xu et al. (10). A sample item is ‘The best way to avoid mistakes is to follow the instructions of senior persons. Lastly, mindful safety practices were evaluated by four items adapted from (29). This evaluates the extent of flight attendants’ proactivity to take action to cope with potential safety hazards and emergencies in the cabin during the flight. A sample item is ‘I stop working if I find that continuing could imply a danger to myself or to others during the flight.’ Comments from the pre-test and pilot test conducted remind the researchers to reconsider the appropriateness of some items in the questionnaire, which needs to be clearly defined and related to the flight attendants’ job context. As a result, some modest modifications have been made to the questions under the constructs of employee voice behavior, psychological safety, traditionality, and mindful safety practices based on the context of research populations and subjects. The items were adjusted to more closely represent flight attendants’ job features and conform to this research context. The scale used to obtain the measures of the variables is a seven-point Likert scale from 1 = very strongly agree to 7 = not at all agree. In addition, the impacts of flight attendants’ age, gender, job tenure, job positions, and marital status have been controlled to avoid statistical confounds.

### Data collection procedure

Back translation involves the comparison between the original questionnaire and the translated version to ensure the accuracy of the translation (30). The original questionnaire is in English version. One bilingual linguist was invited to translate the items from English to Chinese. Further, another bilingual linguist was invited to translate the Chinese items into English version. A comparison was made to make sure there was no ambiguity between these two versions. Before formal questionnaire distribution, the research objectives and process were explained to human resource (HR) managers to get permission for data collection. Researchers and companies reached an agreement on data confidentiality. In all, four private commercial airline companies agreed to take part in data collection. After permission, HR managers helped researchers distribute online questionnaire linkage through the airline’s internal work contact WeChat group. An electrical cover letter was attached before the questionnaire content, which emphasized the study aims and confidentiality of responses. To ensure sufficient variance in the variables in this research model for hypotheses testing, the targeted participants cover different demographic profiles (age, gender, job position, and working experience). The questionnaire was distributed in two timelines. The Time 1 questionnaire contains independent variables (psychological safety), mediators out of order (in-flight safety communication, upward safety communication, and pro-social safety voice), as well as items about demographic profiles. The Time 2 questionnaire consists of the moderators (ethical leadership and traditionality) and dependent variables (mindful safety practices) after 3 weeks. Such data collection practices aim to minimize the potential threat of common method bias (31). After two stages of data collection, the respondents’ answers were then combined and coded.

The questionnaire linkage originates from the Chinese online questionnaire platform called WJX.cn. By WJX.cn, the respondent answers could be posted, tracked, and gathered in real time. All participants filled up the consent form and completed the questionnaire online. The practices of Podsakoff et al. (31) were adopted to reduce the influence of social expectations and CMV (Common method variance). Firstly, the questionnaire was used for research purposes only, and the respondents’ identities were kept anonymous. Secondly, all the respondents’ participation was based on a voluntary perspective, and only real answers were encouraged. Respondents were asked to answer according to what ‘really reflected’ in their experience. This was critical to ensure the truthful answers and real thinking of the respondents. Third, the sequence arrangement of constructs and their items in the questionnaire are disordered with some reverse-coded items as well. Fourth, the data collection was conducted two times with an interval of 3 weeks. Through this time separation, potential CMV could be minimized. Further, the statements of the questionnaire have been made as brief and clear as possible. After this, a pre-test with 10 flight attendants and 2 flight pursers replied that the questionnaire items were easy to understand without ambiguity. Moreover, a pilot test was run to determine the reliability of the items for each construct. When the values of Cronbach’s ɑ are more than 0.7, indicating that the reliability is satisfied. A pilot test was conducted among 33 Chinese flight attendant participants to examine the reliability of the instruments by SPSS Reliability Analysis. The results show that Cronbach’s alpha values are 0.825 (ethical leadership), 0.856 (employee voice behavior), 0.716 (psychological safety), 0.729 (traditionality), and 0.743 (mindful safety practices), indicating a high level of internal consistency for all measures.

### Data analysis

The demographic profiles of the respondents, common method bias, mean and standard deviations for constructs were analyzed using SPSSv29 software. Further, for the research model examination, PLS-SEM was adopted for data analysis and hypothesized relationships that contain direct, mediating, and moderating mechanisms. PLS-SEM is not constrained by the normal distribution of the data as a non-parametric statistical test. The Smart PLS 3.0 software was used to examine the research model. A two-step approach has been taken in terms of measurement and structural model assessments. In the measurement model, factor loading, reliability, convergent, and discriminant validities were all confirmed at first. Further, the structural model was assessed to examine the hypothesized associations among study variables.

## Results

### Response rate

A self-administered questionnaire was used to collect data from flight attendants during the 14 months from late 2022 to early 2024. Of the 1,000 distributed questionnaires, 630 were returned, and 9 invalid questionnaires were discarded, resulting in 621 valid responses with a response rate of 63%.

### Descriptive results

Six hundred and twenty one valid questionnaires were collected from four Chinese private commercial airline companies. Among these respondents, 76.6% of the respondents (*n* = 475) were female while 23.4% of the respondents (*n* = 146) were male. The majority of respondents had less than 10 years of working experience (62%), while 22% of the respondents had 11–15 years of working experience. 46% of the respondents were 18–25 years old, and 32% of the respondents were between 25 and 35 years old. Moreover, from the job position perspective, 73.1% of the respondents were flight attendants (*n* = 454), 18.7% of the respondents (*n* = 116) were deputy pursers, and 51 respondents were pursers. Descriptive statistics were computed for each measurement in the current study. Table 1 demonstrates the mean score and standard deviation for all measures. The mean values for ethical leadership and traditionality are 5.03 and 5.04 respectively, indicating these constructs were nearly good in most of its measurement items. Among the three dimensions of employee voice behavior, in-flight safety communication has the most influence on mindful safety practices adopted by flight attendants.

**Table 1:** Descriptive statistics.

Construct	Mean	Standard deviation
Psychological safety	4.26	1.71
In-flight safety communications	4.30	1.77
Upward safety communications	3.76	1.70
Pro-social safety voice	4.26	1.71
Ethical leadership	5.03	1.52
Traditionality	5.04	1.65
Mindful safety practices	4.21	1.69

Among the three dimensions of employee voice behavior, in-flight safety communication has the most influence on mindful safety practices adopted by flight attendants.

### Common method bias

Since this study relied on self-reported data, it may lead to common method bias (CMB). Therefore, both procedural and statistical methods were employed to mitigate its effects. Harman’s single-factor test revealed that the first factor explained a variance of 38.56%, which is well below the threshold of 50% (31).

### Measurement model assessment

**Figure 2:**
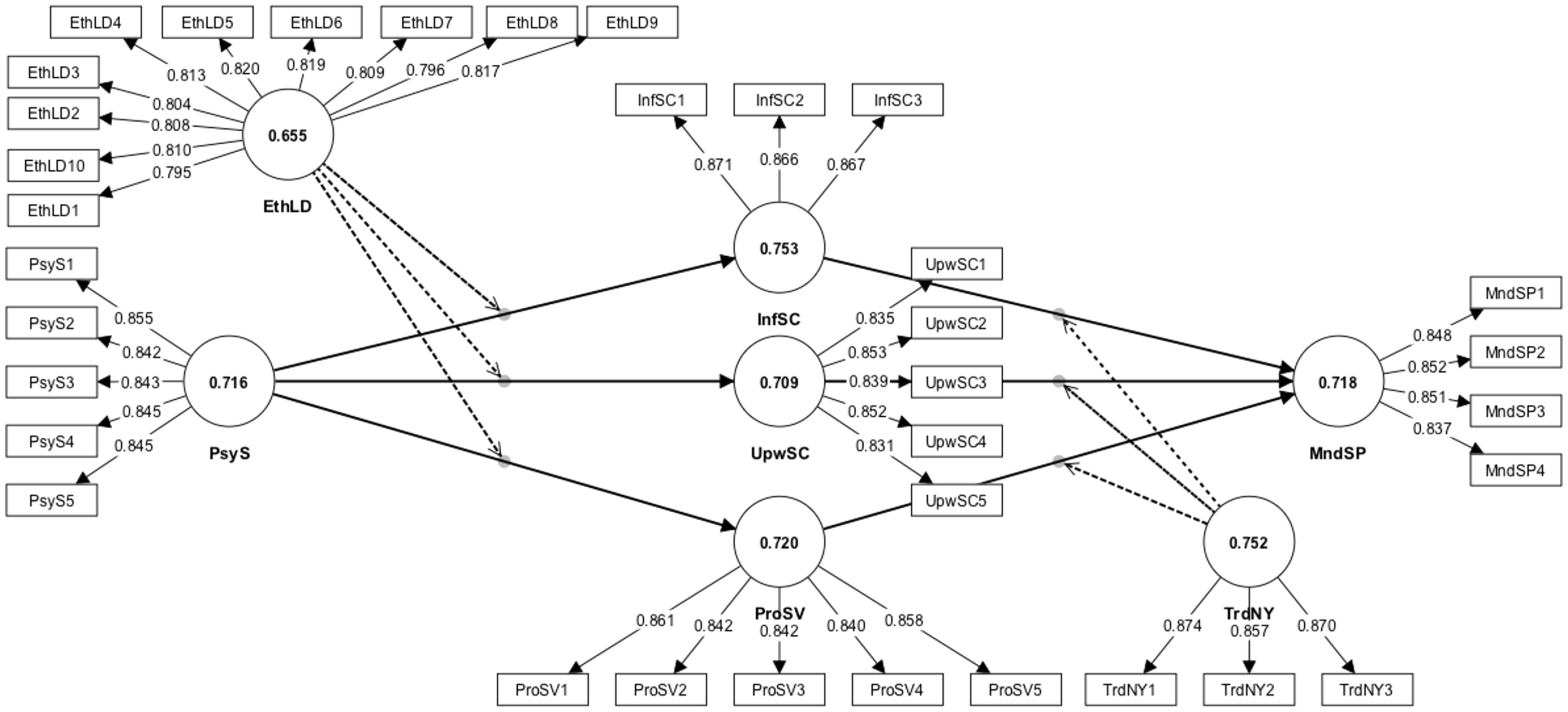
Measurement model assessment. Thus, it can be said that the discriminant validity is established.

The research model of the current study consists of all reflective constructs. Consequently, internal consistency reliability, convergent validity, and discriminant validity were examined as per the guidelines of Hair et al. (32). Cronbach’s ɑ and composite reliability values of all constructs exceeded the threshold of 0.70, which confirms the internal consistency reliability of the data, as shown in Table 2 and Figure 2. Similarly, the factor loadings of all items and average variance extracted (AVE) values of relevant constructs exceeded the 0.708 and 0.50 thresholds, respectively (32).

**Table 2:** Measurement model.

Construct	Item	FL	VIF	CA	CR	AVE
Psychological safety	PsyS1	0.855	2.448	0.901	0.926	0.716
	PsyS2	0.842	2.289			
	PsyS3	0.843	2.220			
	PsyS4	0.845	2.284			
	PsyS5	0.845	2.366			
Ethical leadership	EthLD1	0.795	2.275	0.941	0.950	0.655
	EthLD2	0.808	2.391			
	EthLD3	0.804	2.364			
	EthLD4	0.813	2.425			
	EthLD5	0.820	2.529			
	EthLD6	0.819	2.470			
	EthLD7	0.809	2.377			
	EthLD8	0.796	2.319			
	EthLD9	0.817	2.424			
	EthLD10	0.810	2.437			
In-flight safety communication	InfSC1	0.871	1.891	0.837	0.902	0.753
	InfSC2	0.866	2.009			
	InfSC3	0.867	1.969			
Upward safety communication	UpwSC1	0.835	2.224	0.897	0.924	0.709
	UpwSC2	0.853	2.344			
	UpwSC3	0.839	2.263			
	UpwSC4	0.852	2.387			
	UpwSC5	0.831	2.156			
Pro-social safety voice	ProSV1	0.861	2.463	0.903	0.928	0.720
	ProSV2	0.842	2.312			
	ProSV3	0.842	2.279			
	ProSV4	0.840	2.293			
	ProSV5	0.858	2.480			
Traditionality	TrdNY1	0.874	1.953	0.835	0.901	0.752
	TrdNY2	0.857	1.856			
	TrdNY3	0.870	2.033			
Mindful safety practices	MndSP1	0.848	2.019	0.869	0.910	0.718
	MndSP2	0.852	2.186			
	MndSP3	0.851	2.180			
	MndSP4	0.837	1.979			

*N = 621, FL, factor loading; VIF, variance inflation factor; CA, Cronbach’s ɑ; CR, composite reliability; AVE, average variance extracted. Thus, it can be said that the discriminant validity is established.*

Additionally, the discriminant validity was assessed through the HTMT (Heterotrait-monotrait ratio) values. The results in Table 3 show that all HTMT values were well below the conservative benchmark of 0.85 (33, 34). However, it is worth mentioning that the HTMT value between upward safety communication and pro-social safety voice was observed to be high, even above the liberal threshold of 0.90. In such a situation, Franke and Sarstedt (35) and Hair et al. (32) suggested investigating a 95% one-sided bootstrap confidence interval to check the uniqueness of the constructs. Hair et al. (36) argued that a 95% one-sided bootstrap confidence interval containing the value 1 indicates a deficiency in discriminant validity. Otherwise, it shows empirical distinctiveness between the two constructs. Table 3 demonstrates that the bias-corrected confidence interval does not contain 1 for all combinations of constructs. Thus, it can be said that the discriminant validity is established.

**Table 3:** Discriminant validity.

Construct	EthLD	InfSC	MndSP	ProSV	PsyS	TrdNY	UpwSC
Ethical leadership							
In-flight safety communication	0.556 [0.501, 0.607]						
Mindful safety practices	0.550 [0.496, 0.599]	0.564 [0.505, 0.618]					
Pro-social safety voice	0.501 [0.445, 0.554]	0.52 [0.457, 0.578]	0.597 [0.545, 0.647]				
Psychological safety	0.337 [0.270, 0.402]	0.369 [0.301, 0.436]	0.399 [0.335, 0.464]	0.354 [0.288, 0.418]			
Traditionality	0.514 [0.458, 0.572]	0.556 [0.494, 0.618]	0.554 [0.497, 0.607]	0.515 [0.456, 0.573]	0.339 [0.273, 0.406]		
Upward safety communication	0.489 [0.434, 0.541]	0.543 [0.484, 0.599]	0.576 [0.523, 0.629]	0.984 [0.971, 0.996]	0.350 [0.280, 0.416]	0.507 [0.447, 0.566]	

*N = 621, PsyS, psychological safety; EthLD, ethical leadership; InfSC, in-flight safety communication; UpwSC, upward safety communication; ProSV, pro-social safety voice; TrdNY, traditionality; MndSP, mindful safety practices. Thus, it can be said that the discriminant validity is established.*

### Structural model assessment

The mutual relationships and casualty among the studied variables were evaluated in this phase. However, before proceeding, multicollinearity must be assessed through the variance inflation factor (32). The results in Table 2 show that all VIFs were well below the threshold of 3, indicating the absence of collinearity issues in the data based on Kock (37). Moving forward, a total of 15 hypothesized relationships (six direct, three mediating, and six moderating) were tested using *t* statistics and bootstrapping procedure with 10,000 resampling techniques at a 5% significance level (32).

The pictogram of the structural model is presented in Supplementary Figures S1, S2, while bootstrap findings are tabulated in Table 4. It revealed that psychological safety was positively related to in-flight safety communication (*H_1_*: psychological safety → in-flight safety communication, *β* = 0.321, *t* = 8.858***). Interestingly, psychological safety was found to be negatively associated with upward safety communication (*H_2_*: psychological safety → upward safety communication, *β* = −0.316, *t* = 8.540***). Furthermore, the relationship between psychological safety and pro-social safety voice was found to be positively significant (*H_3_*: psychological safety → pro-social safety voice, *β* = 0.320, *t* = 8.844***). Additionally, H4 demonstrated a significant positive relationship between in-flight safety communication and mindful safety practices (*H_4_*: in-flight safety communication → mindful safety practices, *β* = 0.298, *t* = 8.354***). Conversely, H5 represented a non-significant relationship between upward safety communication and mindful safety practices (*H_5_*: upward safety communication → mindful safety practices, *β* = −0.089, *t* = 1.261^ns^). Lastly, pro-social safety voice was found to be positively related to mindful safety practices (*H_6_*: pro-social safety voice →mindful safety practices, *β* = 0.317, *t* = 4.620***).

**Table 4:** Hypotheses testing.

Relationships	Beta	SD	*t*-value	Decision
Direct				
*H_1_*: Psychological safety → In-flight safety communication	0.321	0.036	8.858***	Accept
*H_2_*: Psychological safety → Upward safety communication	−0.316	0.037	8.540***	Reject
*H_3_*: Psychological safety → Pro-social safety voice	0.320	0.036	8.844***	Accept
*H_4_*: In-flight safety communication → Mindful safety practices	0.298	0.036	8.354***	Accept
*H_5_*: Upward safety communication → Mindful safety practices	−0.089	0.071	1.261^ns^	Reject
*H_6_*: Pro-social safety voice→ Mindful safety practices	0.317	0.069	4.620***	Accept
Mediation				
*H_7_*: Psychological safety → In-flight safety communication → Mindful safety practices	0.096	0.016	5.820***	Accept
*H_8_*: Psychological safety → Upward safety communication → Mindful safety practices	0.028	0.023	1.222^ns^	Reject
*H_9_*: Psychological safety → Pro-social safety voice → Mindful safety practices	0.101	0.026	3.956***	Accept
Moderating				
*H_10_*: Ethical leadership × psychological safety → In-flight safety communication	0.056	0.032	1.714*	Accept
*H_11_*: Ethical leadership × psychological safety → Upward safety communication	−0.019	0.033	0.572^ns^	Reject
*H_12_*: Ethical leadership × psychological safety→ Pro-social safety voice	0.021	0.033	0.632^ns^	Reject
*H_13_*: Traditionality × In-flight safety communication → Mindful safety practices	−0.035	0.037	0.963^ns^	Reject
*H_14_*: Traditionality × Upward safety communication → Mindful safety practices	−0.135	0.065	2.096*	Accept
*H_15_*: Traditionality × Pro-social safety voice → Mindful safety practices	−0.131	0.064	2.055*	Accept

*N* = 621, ***p < 0.001, *p < 0.05; ns, not significant; SD, standard deviations.In contrast, the collective influence of ethical leadership and traditionality only increased the explained variance by 3.8% in mindful safety practices.

For the mediation analysis, three hypotheses, H7, H8, and H9, were proposed and tested using Preacher and Hayes (38) procedure. The results of the first mediation revealed that in-flight safety communication demonstrates a significant positive indirect effect between psychological safety and mindful safety practices (*β* = 0.096, *t* = 5.820***). Conversely, the data of the second mediation does not provide sufficient evidence to conclude that upward safety communication mediates the relationship between psychological safety and mindful safety practices, thus this hypothesis was rejected (*H_8_*: psychological safety → upward safety communication → mindful safety practices, *β* = 0.028, *t* = 1.222^ns^). Finally, the third mediation revealed that pro-social safety voice plays a significant role in linking psychological safety to mindful safety practices (*β* = 0.101, *t* = 3.956***).

**Figure 3:**
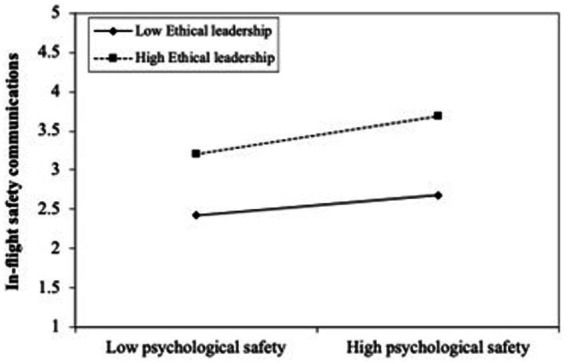
Moderating effect of ethical leadership on the relationship between psychological safety and in-flight safety communication. In contrast, the collective influence of ethical leadership and traditionality only increased the explained variance by 3.8% in mindful safety practices.

**Figure 4:**
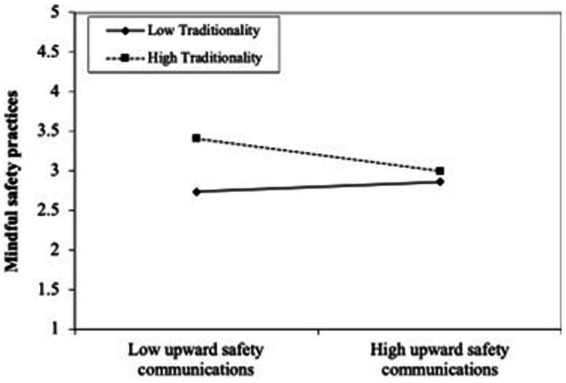
Moderating effect of traditionality on the relationship between pro-social safety voice and mindful safety practices. In contrast, the collective influence of ethical leadership and traditionality only increased the explained variance by 3.8% in mindful safety practices.

Beginning with the moderating effects, the findings revealed that higher levels of ethical leadership strengthened the influence of psychological safety on in-flight safety communication as shown in Figure 3 (*H_10_*: ethical leadership x psychological safety → in-flight safety communication, *β* = 0.056, *t* = 1.714*). However, contrary to the proposed hypotheses, ethical leadership was not found to have a significant interaction effect between psychological safety and upward safety communication (*β* = −0.019, *t* = 0.572^ns^) or pro-social safety voice (*β* = 0.021, *t* = 0.632^ns^). Likewise, the results revealed that traditionality is not a significant moderator on the relationship between in-flight safety communication and mindful safety practices (*H_13_*: traditionality × in-flight safety communication → mindful safety practices, *β* = −0.035, *t* = 0.963^ns^). Lastly, both H14 and H15 reveal significant negative interaction effects and also indicate that higher levels of traditionality weaken the positive impact of upward safety communication as demonstrated by Figure 4 (*H_14_*: traditionality × upward safety communication→ mindful safety practices, *β* = −0.135, *t* = 2.096*) and pro-social safety voice (*H_15_*: traditionality × pro-social safety voice → mindful safety practices, *β* = −0.131, *t* = 2.055*).

Despite hypotheses testing, Hair et al. (32) suggested evaluating the effect size (*f*^2^) and explaining the variance (*R*^2^) of the model. Regarding effect size, Cohen (2013) reported three categories of *f*^2^, which are 0.02 (small), 0.15 (medium), and 0.35 (high). The effect size findings of the current study ranged from 0.002 to 0.245, indicating a spectrum from small to medium effect sizes. Meanwhile, the finding also showed that psychological safety explained a variance of 10.3, 10, and 10.2% in in-flight safety communication, upward safety communication, and pro-social safety voice, respectively. In this study, the results show that the dependent variable (mindful safety practices) explains 35.8% of the variance of the structure model.

However, the inclusion of ethical leadership and traditionality as moderators, resulted in increased explained variances of all constructs. For example, ethical leadership accounts for an additional 17.7% of the variance in in-flight safety communication, 13.8% in upward safety communication, and 14.8% in pro-social safety voice. In contrast, the collective influence of ethical leadership and traditionality only increased the explained variance by 3.8% in mindful safety practices.

### PLS-predict

The predictive relevance of the model was evaluated through PLS-predict. PLS-predict assesses how well the model forecasts the values of a target construct and its indicators in a new dataset (39). According to Shmueli et al. (40), the *Q*^2^ value above zero is critical in achieving predictive relevance for a specific dependent variable. However, PLS-SEM ≤ LM should be evaluated to check the strength of predictive relevance. It is categorized into high, moderate, weak, or no predictive relevance. Table 5 indicates the results of PLS-predict with and without moderators of the current study. Both models have Q^2^ values above 0, which indicates that predictive relevance exists. Moreover, PLS-SEM ≤ LM was evaluated for root mean square error (RMSE) to check the strength of predictive relevance. Table 5 shows that without a moderator (i.e., ethical leadership), in-flight safety communication, upward safety communication, and pro-social safety voice demonstrate high predictive relevance. Furthermore, mindful safety practices show low predictive relevance without the moderator (i.e., traditionality), but exhibit a high predictive relevance when traditionality (a moderator) is present.

**Table 5:** PLS-predict.

Construct	Item	Without moderator			With moderator		
		Q^2^predict	PLS-SEM_RMSE	LM_RMSE	Q^2^predict	PLS-SEM_RMSE	LM_RMSE
In-flight safety communication	InfSC1	0.080	1.985	1.997	0.218	1.831	1.830
	InfSC2	0.066	1.947	1.956	0.193	1.810	1.778
	InfSC3	0.077	1.947	1.953	0.202	1.810	1.765
Upward safety communication	UpwSC1	0.062	1.963	1.973	0.144	1.875	1.876
	UpwSC2	0.077	1.904	1.911	0.183	1.790	1.785
	UpwSC3	0.068	1.915	1.922	0.158	1.821	1.796
	UpwSC4	0.065	1.968	1.976	0.166	1.858	1.862
	UpwSC5	0.068	1.981	1.984	0.160	1.880	1.838
Pro-social safety voice	ProSV1	0.064	1.995	2.005	0.183	1.864	1.842
	ProSV2	0.076	1.945	1.955	0.172	1.841	1.840
	ProSV3	0.075	1.919	1.929	0.166	1.822	1.791
	ProSV4	0.067	1.924	1.935	0.167	1.817	1.809
	ProSV5	0.074	1.914	1.922	0.181	1.800	1.788
Mindful safety practices	MndSP1	0.080	1.905	1.906	0.229	1.744	1.754
	MndSP2	0.074	1.931	1.935	0.224	1.768	1.790
	MndSP3	0.077	1.935	1.936	0.242	1.754	1.760
	MndSP4	0.074	1.895	1.894	0.216	1.744	1.753

*N = 621, PLS-SEM_RMSE, partial least square- structural equation modelling _ root means square error; LM_RMSE, linear model _ root means square error.*

## Discussion

Despite the importance of human error and many underlying factors that could predict the safety performance of flight attendants in the airline industry. However, less academic attention has been paid to the underlying mechanism through which employee safety voice and voluntary safety-related behaviors could be triggered. Against this backdrop, this study developed and tested a conceptual model that proposed three types of employee voice behaviors as proximal mediating factors in the relationship between psychological safety and mindful safety practices. The results demonstrate the validity of the conceptual model proposed and prove several hypotheses developed in the current study. According to the results, psychological safety is a greater indicator of flight attendants’ in-flight safety communication and pro-social safety voice. More importantly, these findings advanced existing knowledge regarding the association between employee psychological status and their safety voice behavior. In addition, mindful safety practices have been chosen as a type of voluntary and extra-role safety behavior, which seems to be prevalent in several high-risk industries, including the aviation industry. Furthermore, in-flight safety communication and pro-social safety voice have been incorporated in the present study as two effective voice behaviors among flight attendants to promote mindful safety practices adoption in the aviation industry. In light of SET, and COR theories, the results found that ethical leadership enhances the relationship between psychological safety and flight attendants’ in-flight safety communication. Nevertheless, traditionality weakens and reduces the effective impacts of upward safety communication and pro-social safety voice on mindful safety practices adoption.

### Theoretical implications

This study finds that psychological safety plays an important role in predicting flight attendants’ voice behavior. This finding is in line with some previous studies that identified a positive link between subordinates’ perceived psychological safety and their voice behavior (41–43). Previous research (44) found that upward safety communication has a positive and significant impact on both safety compliance and proactive safety performance of flight attendants. However, the proposed positive relationship between upward safety communication and mindful safety practices adopted by flight attendants in this research was rejected. Two types of voice behavior (in-flight safety communication and pro-social safety voice) were found as important antecedents of flight attendants’ mindful safety practices adoption in this research. The application of employee voice participation provides a novel direction for mindful safety practices facilitation in the area of aviation safety. Moreover, the results add to the ongoing debate in the safety literature by addressing both positive and negative impacts on flight attendants’ voice behavior. Previous research by Chen (13) found that morality leadership could lead to flight attendants’ pro-social voice behavior, which is different from the positive role of ethical leadership in enhancing flight attendants’ in-flight safety communication in this study. Moreover, Mathisen et al. (8) found that leader support is a resourceful variable that could strengthen employees’ safety voices and buffer the negative effects of job demands in the high-risk industry. This research proves how ethical leadership enhances the relationship between flight attendants’ psychological safety and in-flight safety communication. Moreover, traditionality exerts a diminishing effect on flight attendants’ upward safety communication and pro-social voice in their mindful safety practices implementation. The results indicate that in organizations with a high level of traditionality, the association between employee voice behavior and mindful safety practices adoption appears to be weaker than in organizations with a low level of traditionality.

Through interviewing some flight attendants working for two Chinese private commercial airline companies, potentially possible causes of the above research results were identified as follows. Conducting upward safety communication is still difficult for Chinese employees due to the traditionality and culture in the organizations. Therefore, compared to the other two types of voice behaviors, employees usually do not choose to conduct upward safety communication in the Chinese work context. Moreover, flight attendants usually interact and communicate with their direct supervisors on the same flights. So ethical leadership plays a more important and strengthening role in the relationship between flight attendants’ psychological safety and in-flight safety communication. On account of the significance of safety and life issues during flight duties, in-flight safety communication from the flight attendants is not affected by traditionality as a life-and-death matter. That indicates that in-flight safety communication was less constrained by external factors during flight tasks.

### Practical implications

Psychological safety has an indirect effect on flight attendants’ safety performance (mindful safety practices) through two types of voice behaviors (in-flight safety communication and pro-social safety voice). Therefore, this finding acknowledges the significance of psychological safety, as a positive psychological status of employees would have managerial implications in aviation companies. From a practical perspective, our findings indicate psychological safety and flight attendants’ voice behaviors may provide feasible ways to enhance the initiative employee safety performance. Also, flight attendants with a high level of psychological safety would be more likely to conduct voice behavior, which further contributes to extra-role safety performance.

How can we encourage flight attendants to participate in safety-related voice activities? The present study indicates that ethical leadership could strengthen the link between flight attendants’ psychological safety and in-flight safety communication. The finding suggests that supervisors ought to adopt the ethical leadership style during flight duties. Suitable leadership style selection and adoption foster a communication-friendly working environment. Bienefeld and Grote (45) found that the main reasons associated with flight attendants’ silence are fear of punishment, a sense of futility, and concern about damaging relationships At the organizational level, organizations ought to ensure policies, processes, and procedures established to encourage employees’ voluntary safety reporting by a suitable organizational culture (19). Front-line employees with psychological safety are more willing to discuss errors and potential safety problems in a threat-free, open, and no-blaming work environment (41, 46). Moreover, supervisors and organizations need to protect the employees’ confidentiality after their voice behavior for only organizational safety improvement purposes (19).

Additionally, the failure of teamwork and communication between the flight attendants and pilots is also one of the main reasons for flight accidents. It stresses the necessity of crew resource management (CRM) training programs for flight attendants (5). CRM training is an effective way for both flight attendants and pilots to improve teamwork and communication (25). The present study points out the hindering role of traditionality between flight attendants’ two types of voice behaviors (upward safety communication and pro-social voice) and mindful safety practices adoption. Manapragada and Bruk-Lee (47) discovered that employees’ motives for remaining silent about safety issues in the workplace may come from different factors, like organizational climate and job perspectives. Thus, psychological safety highly depends on the working environment, which should reduce the traditional hierarchy level to encourage brainstorming and put flight attendants and pilots together to gain more solutions for in-flight emergency scenarios.

### Limitations and future research direction

The data was collected from flight attendants in the Chinese aviation industry, which negatively affects the generalizability of findings to other high-risk industries and respondents from different job positions. Thus, future research should target different job positions and cultures. Secondly, this study was conducted by a cross-sectional approach at a single point in time. Hence, future researchers could try a longitudinal research design to track the dynamics of flight attendants’ mindful safety practices facilitation over time. Thirdly, the research inevitably has common method variance and social desirability bias during the data collection process. Lastly, we concluded that flight attendants’ psychological safety is not directly related to mindful safety practices application, but mediated through voice behavior. It is suggested that future researchers could explore the effects of more potential mediations between psychological safety and employee-mindful safety practices adoption.

## Data Availability

The raw data supporting the conclusions of this article will be made available by the authors, without undue reservation.

## Supplementary material

SUPPLEMENTARY FIGURE S1 Structural model assessment (without moderator).

SUPPLEMENTARY FIGURE S2 Structural model assessment (with moderator).
