# Supplementary material for: The effects of psychological safety and employee voice behavior on flight attendants’ mindful safety practices adoption
Source: Front Public Health. 2024 Sep 11;12:1398815. doi: 10.3389/fpubh.2024.1398815 (PMC11422219; doi:10.3389/fpubh.2024.1398815)
Supplement: SUPPLEMENTARY FIGURE S1 — Structural model assessment (without moderator). [file Data_Sheet_1.doc]

**Supplementary Material**

This section supplies the theoretical foundation of the conservation of resources (COR) theory and social exchange theory (SET). Furthermore, the hypotheses development covering the relationships among independent variables, mediators, moderators, and dependent variables are described in more detail in this section.

**Theoretical Foundation**

**Conservation of Resources (COR) Theory**

Additionally, encouraging employees to express ideas openly is also considered as one crucial organizational resource (Hobfoll, 2011). Following COR theory, ethical leadership can prompt the enhancement and fit between psychological safety and employee voice behavior. It is argued that direct supervisors frequently interact with subordinates in the daily work routine by assigning, monitoring, and evaluating their jobs (Xu et al., 2019). Hence, leadership adoption as an imperative condition resource plays a vital role in supporting and shaping employee work behavior. Furthermore, adopting an ethical leadership style can establish a safe working environment for employees to express their ideas as two-way communication is encouraged (Brown et al., 2005). Employee psychological safety as a personal resource enables individuals to proactively contribute to more work engagements and involvements (Xu et al., 2022).

**Social Exchange Theory (SET）**

This interchange would be demonstrated through elevated levels of employee work performance, such as voice participation. By strengthening this relationship, SET provides guidelines about the moderating effect of ethical leadership between psychological safety and three types of employee voice participation. The rules of exchange and repaying involved in SET induce the employees to feel obligated and indebted to their organizational goal achievement (Cropanzano and Mitchell, 2005). Through SET, the relationship between the supervisors and subordinates could be enhanced under mutual trust and closed relational bonds.

**Hypotheses Development**

**Psychological Safety and Employee Voice Behavior**

Team psychological safety reflects team members' shared and implied beliefs at a group level (Edmondson, 1999). Under the SET, group members with psychological safety are more willing to put effort and engagement into organizational improvements by voice behavior (Rees et al., 2013). Employee’s safe perceptions of speaking up about errors and problems facilitate organizational learning behavior, and learning from failures without blaming others (Carmeli and Gittell, 2009). Under a psychologically safe organizational climate, employees can achieve communication across organizational hierarchy status and role boundaries (Edmondson et al., 2004; Taggar, 2002). This type of information sharing among the team members also prompts knowledge and idea sharing (Edmondson & Lei, 2014). Employees with psychological safety trust in their colleagues, managers, and organizations (Ahmad et al., 2022).

Employee voice contains ideas, suggestions, concerns, and opinions about organizational issues, aiming to improve organizational performance in a discretionary way (Morrison, 2011). Employee safety voice participation reflects their willingness to communicate safety-related work issues with their colleagues and leaders (Chen, 2017). Liang et al. (2012) discussed the two categories of employee voice from prohibitive and promotive perspectives, covering the ideas or suggestions for organizational improvements or harm prevention. Rees et al. (2013) said that voice practices could be achieved through a two-way flow, inducing interactions from the mutual sides (employers and employees). Employees feel obligated and motivated to conduct voice behavior when organizations encourage them to participate in organizational improvement and development (Shin et al., 2022). Employee upward safety communication reflects employee willingness to speak up about new ideas and suggestions related to safety with their department managers. In-flight safety communication refers to the communication between flight attendants and other cabin crews during flight missions. Additionally, employees with pro-social voice behavior tend to provide constructive recommendations to benefit whole organizational performance, considering as the most effective voice behavior (Van Dyne et al., 2003).

**Employee Voice Behavior and Mindful Safety Practices**

Weick et al. (2008) integrated collective mindfulness as a shared mental process into high-reliability operations. However, mindful safety practices reflect individual mindfulness, aiming to prevent and intervene in unanticipated events based on their abilities and subjective judgments (Aase et al., 2005). The subsets of mindful safety practices contend that employees not only pay attention to their own operations but also warn and care for colleagues about potential dangers during work (Skjerve, 2008). It is discussed that mindful safety practices are more closed to safety-promoting programs with the focus of unwanted and unanticipated event prevention (Skjerve, 2008). It is difficult for mindful safety practices to be required as a formal organizational behavior as it needs employees’ subjective judgments, awareness, and appropriate reactions to potential dangers (Dahl and Kongsvik, 2018).

In some situations, employees intentionally withhold their opinions even if their ideas are constructive and beneficial for organizational performance. This silent behavior harms organizational development as it can bring more ambiguities and prevent improving opportunities for organizations. Voice behavior, especially pro-social voice behavior, employees can constructively express ideas, information, and opinions to benefit organizational performance (Van Dyne et al., 2003).

**Mediating Role of Employee Voice Behavior and Mindful Safety Practices**

Mindful safety practices are a series of comprehensive actions involving risk anticipation, problem-solving, decision-making, and appropriate actions to mitigate potential hazards and risks in the workplace. Flight attendants with psychological safety are more likely to voluntarily report inappropriate safety procedures and human errors, which further facilitates mindful safety practices adoption. Thus, this research hypothesizes flight attendants with psychological safety would have enough motivation to express their ideas, observations, and constructive recommendations about safety-related issues in the workplace.

**Moderating Role of Ethical Leadership and Employee Voice Behavior**

Ethical leadership (ETL) is defined as the leaders’ normative and appropriate conduction through their ethical actions, interpersonal relationships, and behavioral promotion to followers through two-way communication, moral reinforcement, and ethical decision-making process (Brown et al., 2005). Leadership style is an essential antecedent for employee psychological safety as leaders demonstrate how they respect and value the subordinates through leadership style selection (Yin et al., 2020; Edmondson.,1999). The main characteristic of ethical leadership is ethical orientation and leaders behave as a role model in the decision-making process and daily work operations (Thun & Kevin Kelloway, 2011; Charoensap et al., 2019). Supervisors with ethical leadership usually give an ear to, show consideration, and care for subordinates (Brown et al., 2005; Toor & Ofori, 2009). Ethical leadership adoption also promotes open communication in the work environment because this leadership style encourages two-way communication. Along with this, a high level of ethical leadership facilitates the subordinates to go beyond the primary job duties and achieve more extra-role job performance (Colquitt et al., 2007). Eluwole et al. (2022) found that ethical leadership could foster employees' trust in the organization. In the Chinese tradition, leaders with a high level of personal virtues and morality as role models are highly trusted and respected by subordinates (Chen, 2017). Under this leadership style, mutual interactions and sharing between leaders and subordinates could facilitate optimal problem solutions in the workplace (Huang & Liu, 2022). By voluntary voice behavior, flight attendants with high self-efficacy could better contribute to improving organizational performance. Dedahanov et al.(2016) have identified moral leadership as one of the critical antecedents for employee voice behavior prediction. Chen (2017) also found that morality leadership significantly affects cabin crews' willingness to conduct upward and pro-social voice behavior in the aviation industry.

**Moderating Role of Traditionality and Mindful Safety Practices**

Traditionality reflects the citizen’s accepting situation of traditions and customs in a society (Schwartz, 1992). Yang et al. (1989) divided the Chinese traditionality construct into five dimensions, such as obedience to authority, conservation and patience, fatalism and defense. Among these five dimensions, authority obedience is highly related to organizational and management activities in Chinese society. Employees with high traditionality tend to fulfill sound job expectations, responsibilities, and involvement due to solid social role identification (Farh et al., 2007; Xu et al., 2022). Nevertheless, everything has two sides, just like coins. On one hand, an employee with a high level of traditionality is more likely to complete job tasks. Xu et al. (2022) said that the greatest advantage of traditionality is that employees choose to obey to fulfill their social roles and job responsibilities, leading to more job involvement. On the other hand, this hierarchy concept highly emphasizes the leaders’ authority with less employee autonomy. A high-level hierarchical structure as an institutional factor prevents the employees’ intentions to conduct voice behavior (Mathisen et al., 2022). China has a high uncertainty avoidance and power distance culture based on the cultural classifications by Hofstede (2001). Under this culture, employees can bear unequal power distribution and misuse to a certain degree compared to the culture with low uncertainty avoidance and power distance. It is argued that under the high uncertainty avoidance culture, leaders usually tend to give more control and less autonomy to their subordinates (Hofstede, 2001). According to Xu et al. (2022), traditionality as a personality and custom is relatively stable and long-standing. Additionally, GuanXi is a Chinese English word, indicating the relationship orientation among the people in Eastern culture (Chen et al., 2013; Yen et al., 2011). In Chinese traditionality, individuals prefer a long-term established GuanXi than an immediate trade-off in dealing with work issues (Lv et al., 2022). Employees with high traditionality are more likely to comply with authority and follow a hierarchical organizational system and structure (Hui et al., 2007).

**References**

Aase, K., Skjerve, A. B. M. and Rosness, R. 'Why good luck has a reason: mindful practices in offshore oil and gas drilling'. *International Conference on Organizational Learning and Knowledge*, Trento: University of Trento, 2005, 193-210.

Ahmad, N., Ullah, Z., AlDhaen, E., Han, H. and Scholz, M. (2022) 'A CSR perspective to foster employee creativity in the banking sector: The role of work engagement and psychological safety', *Journal of Retailing and Consumer Services,* 67, pp. 102968.

Carmeli, A. and Gittell, J. H. (2009) 'High-quality relationships, psychological safety, and learning from failures in work organizations', *Journal of Organizational Behavior,* 30(6), pp. 709-729.

Charoensap, A., Virakul, B., Senasu, K. and Ayman, R. (2019) 'Effect of Ethical Leadership and Interactional Justice on Employee Work Attitudes', *Journal of Leadership Studies,* 12(4), pp. 7-26.

Colquitt, J. A., Scott, B. A. and LePine, J. A. (2007) 'Trust, trustworthiness, and trust propensity: A meta-analytic test of their unique relationships with risk taking and job performance', *Journal of applied psychology,* 92(4), pp. 909-927.

Cropanzano, R. and Mitchell, M. S. (2005) 'Social Exchange Theory: An Interdisciplinary Review', *Journal of Management,* 31(6), pp. 874-900.

Dahl, Ø. and Kongsvik, T. (2018) 'Safety climate and mindful safety practices in the oil and gas industry', *Journal of Safety Research,* 64, pp. 29-36.

Dedahanov, A. T., Lee, D. H., Rhee, J. and Yoon, J. (2016) 'Entrepreneur’s paternalistic leadership style and creativity: The mediating role of employee voice', *Management Decision,* 54(9), pp. 2310-2324.

Edmondson, A. (1999) 'Psychological Safety and Learning Behavior in Work Teams', *Administrative Science Quarterly,* 44(2), pp. 350-383.

Farh, J.-L., Hackett, R. D. and Liang, J. (2007) 'Individual-Level Cultural Values as Moderators of Perceived Organizational Support–Employee Outcome Relationships in China: Comparing the Effects of Power Distance and Traditionality', *Academy of Management Journal,* 50(3), pp. 715-729.

Hofstede, G. (2001) *Culture's consequences: Comparing values, behaviors, institutions and organizations across nations.* sage.

Huang, C.-Y. and Liu, Y.-C. (2022) 'Influence of need for cognition and psychological safety climate on information elaboration and team creativity', *European Journal of Work and Organizational Psychology,* 31(1), pp. 102-116.

Hui, C., Wong, A. and Tjosvold, D. (2007) 'Turnover intention and performance in China: The role of positive affectivity, Chinese values, perceived organizational support and constructive controversy', *Journal of Occupational and Organizational Psychology,* 80(4), pp. 735-751.

Liang, J., Farh, C. I. C. and Farh, J.-L. (2012) 'Psychological Antecedents of Promotive and Prohibitive Voice: A Two-Wave Examination', *Academy of Management Journal,* 55(1), pp. 71-92.

Lv, W. Q., Shen, L. C., Tsai, C.-H., Su, C.-H., Kim, H. J. and Chen, M.-H. (2022) 'Servant leadership elevates supervisor-subordinate guanxi: An investigation of psychological safety and organizational identification', *International Journal of Hospitality Management,* 101, pp. 103114.

Morrison, E. W. (2011) 'Employee Voice Behavior: Integration and Directions for Future Research', *Academy of Management Annals,* 5(1), pp. 373-412.

Rees, C., Alfes, K. and Gatenby, M. (2013) 'Employee voice and engagement: connections and consequences', *The International Journal of Human Resource Management,* 24(14), pp. 2780-2798.

Schwartz, S. J. (1992). Universals in the content and structure of values: Theory and empirical tests in 20 countries. In M. Zanna (Ed.), *Advances in experimental social psychology* (pp. 1–65). Academic Press

Shin, D., Woodwark, M. J., Konrad, A. M. and Jung, Y. (2022) 'Innovation strategy, voice practices, employee voice participation, and organizational innovation', *Journal of Business Research,* 147, pp. 392-402.

Taggar, S. (2002) 'Individual Creativity and Group Ability to Utilize Individual Creative Resources: A Multilevel Model', *Academy of Management Journal,* 45(2), pp. 315-330.

Thun, B. and Kevin Kelloway, E. (2011) 'Virtuous Leaders: Assessing Character Strengths in the Workplace', *Canadian Journal of Administrative Sciences / Revue Canadienne des Sciences de l'Administration,* 28(3), pp. 270-283.

Toor, S.-u.-R. and Ofori, G. (2009) 'Ethical Leadership: Examiningthe Relationships with Full Range Leadership Model, Employee Outcomes, and Organizational Culture', *Journal of Business Ethics,* 90(4), pp. 533-547.

Weick, K. E., Sutcliffe, K. M. and Obstfeld, D. (2008) 'Organizing for high reliability: Processes of collective mindfulness.', *Crisis management,* 3(1), pp. 81–123.

Yang, K. S., Yu, A. and Yeh, M. H. 'Chinese individual modernity and traditionality: Construct definition and measure'. 1989, 145-169.

Yen, D. A., Barnes, B. R. and Wang, C. L. (2011) 'The measurement of guanxi: Introducing the GRX scale', *Industrial Marketing Management,* 40(1), pp. 97-108.

Yin, J., Ma, Z., Yu, H., Jia, M. and Liao, G. (2020) 'Transformational leadership and employee knowledge sharing: explore the mediating roles of psychological safety and team efficacy', Journal of Knowledge Management, 24(2), pp. 150-171.

**Supplementary Figures**

**
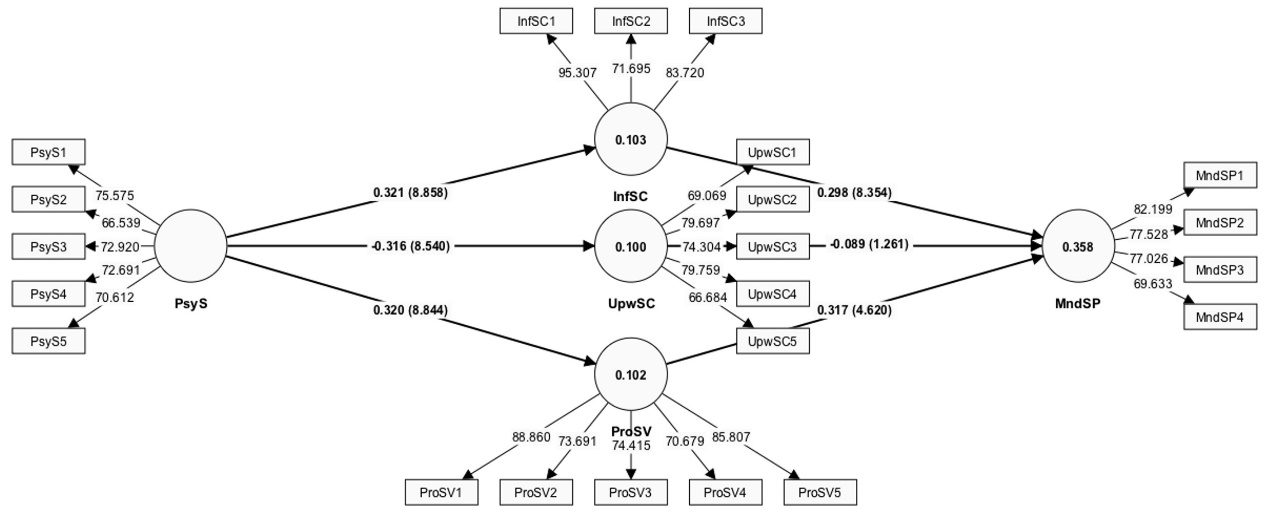
**

Figure S1. Structural Model Assessment (without moderator)


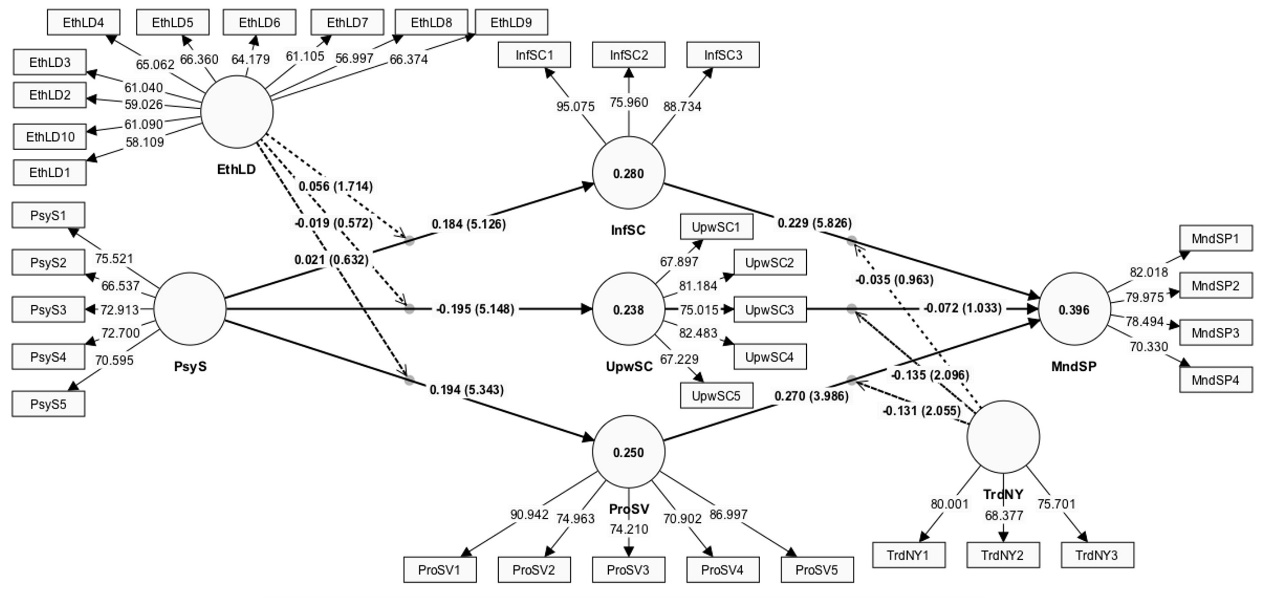


Figure S2. Structural Model Assessment (with moderator)
